# An update of the salmon louse (*Lepeophtheirus salmonis*) reference genome assembly

**DOI:** 10.1093/g3journal/jkac087

**Published:** 2022-04-11

**Authors:** Jay Joshi, Anne-Marie Flores, Kris A Christensen, Hollie Johnson, Ahmed Siah, Ben F Koop

**Affiliations:** 1 Department of Biology, University of Victoria, Victoria, BC V8W 2Y2, Canada; 2 British Columbia Centre for Aquatic Health Sciences, Campbell River, BC V9W 2C2, Canada

**Keywords:** sea lice, Pacific, farmed salmon, ocean, parasite, ZW sex-determination

## Abstract

Salmon lice have plagued the salmon farming industry and have negatively impacted salmon populations in the wild. In response, researchers have generated high density genetic maps, genome assemblies, transcriptomes, and whole-genome resequencing data to better understand this parasite. In this study, we used long-read sequencing technology to update the previous genome assemblies of Atlantic Ocean salmon lice with a more contiguous assembly and a more comprehensive gene catalog of Pacific Ocean salmon lice. We were also able to further characterize genomic features previously identified from other studies by using published resequenced genomes of 25 Atlantic and 15 Pacific salmon lice. One example was further characterizing the ZW sex chromosomes. For both the Atlantic and Pacific Ocean salmon lice subspecies, we found that the female W-chromosome is only a small fraction of the Z-chromosome and that the vast majority of the W and Z-chromosome do not contain conserved regions (i.e. pseudoautosomal regions). However, conserved orthologous protein sequences can still be identified between the W- and Z-chromosomes.

## Introduction 

Salmon lice are small crustaceans belonging to the *Lepeophtheirus* genus of parasitic copepods (Jones and Beamish 2011). These ectoparasites cause economic losses to the aquaculture industry (Costello 2009; Abolofia *et al.* 2017) by feeding on host mucus, skin, and muscle tissues—reducing growth and increasing the chances of mortality in farmed salmon and trout (Thorstad *et al.* 2015). Salmon lice can also negatively impact wild salmon near farms with active infestations (e.g. Krkošek and Hilborn 2011). Off the coast of British Columbia, multiple species of sea lice, often in large numbers, can be found on almost every Pacific salmon of all species (Beamish *et al.* 2005).

Several studies have previously generated genomic resources and characterized some of the major genomic features of salmon lice. Multiple genome assemblies have been produced of the Pacific and Atlantic allopatric subspecies of the salmon louse (Skern-Mauritzen *et al.* 2014, 2021; Messmer *et al.* 2018). Genome assemblies available on NCBI (as of writing) range from a contig N50 of 10–485 kb, and only one is considered a chromosome-level assembly (excluding the current study). In addition, high-density genetic maps were previously produced for the Atlantic subspecies of salmon louse (Besnier *et al.* 2014; Danzmann *et al.* 2019). From these studies, researchers found that salmon lice have 15 pairs of chromosomes and a ZW sex-determination system (Carmichael *et al.* 2013; Besnier *et al.* 2014; Danzmann *et al.* 2019; Skern-Mauritzen *et al.* 2021).

In this study, we produced a new reference genome assembly for salmon lice using third-generation sequencing technology to improve upon existing short-read based genome assemblies. This was done to increase genome contiguity (now 4,500 kb), reduce the number of missing or partial genes, allow standard annotation by the NCBI, and further characterize previously discovered genomic features among the populations sampled.

## Materials and methods

### Sequencing and genome assembly

Multiple Pacific Ocean female salmon lice were collected by members of the British Columbia Centre for Aquatic Health Sciences from an Atlantic salmon farm in March 2020 near West Vancouver Island in British Columbia and flash frozen on dry ice until they could be stored at −80°C. We extracted high molecular weight (HMW) DNA from the cephalothorax of several salmon lice using a modified HMW extraction protocol of the Nanobind Tissue Big DNA Kit [HMW (50–400+ kb) DNA Extraction from Sea Lice homogenized with Pellet Pestle—Protocol 1] (Circulomics). This protocol required the Nanobind Tissue Big DNA Kit (Circulomics) and Buffer PL1 (Circulomics). Following DNA extraction, we used the Short Read Eliminator Kit (Circulomics) to reduce the number of small DNA fragments following the manufacturer’s protocol. Sequencing libraries were prepared according to the manufacturer’s protocol using the Ligation Sequencing Kit (SQK-LSK109 Oxford Nanopore Technologies) and sequenced on a Flow Cell MK1 R9 of a MinION (Oxford Nanopore Technologies). Sequences were generated in FASTQ format using the Guppy Basecalling Software (version 3.4.3+f4fc735).

The initial assembly was then generated using the Flye genome assembler (version 2.7b-b1528) (Kolmogorov *et al.* 2019) with default settings, except for genome-size was set to 0.8G and asm-coverage was set to 50. We used Racon (version 1.4.16, parameters: -u) (Vaser *et al.* 2017) to generate consensus sequences and polish the genome using the Nanopore reads aligned to the assembly with Minimap2 (version 2.13, parameters: -x map-ont) (Li 2018). Pilon (version 1.22) (Walker *et al.* 2014) was used for final polishing with default settings [reads were aligned using the bwa mem program with the parameter -M (Li 2013)]. The reads for this final polishing came from the NCBI SRA database (SRR13076813) and were trimmed using Trimmomatic (version 0.39) (Bolger *et al.* 2014) with the following parameters: ILLUMINACLIP: TruSeq2-PE.fa: 2:30:10:2: keepBothReads, LEADING: 28, TRAILING: 28, MINLEN: 50, TOPHRED33. Chromonomer (version 1.10) (Catchen *et al.* 2020) was used to map contigs onto linkage groups using a genetic map (Messmer *et al.* 2018; Danzmann *et al.* 2019) and the disable_splitting parameter. Only an Atlantic salmon lice subspecies genetic map was available for placing the Pacific salmon lice contigs onto chromosomes, but the assembly can be changed once a Pacific genetic map or Hi-C data becomes available.

### Nucleotide variant calling on previously resequenced genomes

Nucleotide variants were called using resequenced genomes from 25 Atlantic and 15 Pacific salmon lice used in a previous study (Messmer *et al.* 2018) with the methodology presented in Christensen *et al.* (2020) and modified by only using a single truth set for recalibration (see Messmer *et al.* 2018; Danzmann *et al.* 2019, for how markers were generated). Only variants that were successfully used in generating the genetic map from Danzmann *et al.* (2019) were used as the truth set. Briefly, GATK (McKenna *et al.* 2010; DePristo *et al.* 2011; Van der Auwera *et al.* 2013) 3.8 was used to call nucleotide variants by aligning reads to a reference genome, identifying PCR duplicates, calling variants individually, calling variants for all individuals, and finally recalibrating called nucleotide variant scores using known variants. For all analyses, except for the number of missing variants, the nucleotide variants were filtered so only bi-allelic SNPs were used if they were not missing in more than 10% of the samples and had a minor allele frequency of 0.05 or greater using vcftools (version 0.1.15) (Danecek *et al.* 2011). Python scripts (github.com/KrisChristensen/VCFStatistics; last accessed April 18, 2022) were used to process the VCF file to generate all of the data for the Circos plot (version 0.69-9) (Krzywinski *et al.* 2009), except for repetitive elements (github.com/KrisChristensen/NCBIGenomeRepeats; last accessed April 18, 2022). A PCA analysis was performed using PLINK (version v1.90b6.15) (Chang *et al.* 2015) with variants on the chromosomes and visualized using ggplot2 (Wickham 2016) in R (R Core Team 2020).

To identify a list of W-chromosome scaffolds, missing genotypes were compared between male and female lice using a Python script (github.com/KrisChristensen/VCFStatistics) in 10 kb windows (or smaller if the contig was smaller than 10 kb). If there were twice the average number of missing genotypes between male and females, the contigs were manually checked and verified (Supplementary File 1). Nucleotide diversity within (pi) Pacific and Atlantic salmon lice subspecies, as well as between (Dxy) subspecies, was calculated in 10 kb windows using the R package PopGenome (Pfeifer *et al.* 2014) and visualized with qqman (Turner 2018).

## Results and discussion

The chromosome-level reference genome assembly produced in this study is more contiguous and has more gene annotations than other salmon lice assemblies Table 1, (Skern-Mauritzen *et al.* 2021)]. The assembly contiguity increased from a contig N50 of 0.5 Mb (next most contiguous assembly) to 4.5 Mb. The gene count increased from 13,081 (the only other annotated assembly) to 19,181, and the reference genome assembly now has gene annotation generated by the NCBI using a standardized methodology.

**Table 1.** Genome assembly metrics.

Genome	Current	Recently published	Second longest contig N50
	GCF_016086655.3	***Skern-Mauritzen et al.* (** 2021 **)**	GCA_905330665.1
Size	647 Mb^*^	695 Mb	632 Mb^*^
Contigs	8,671^*^	—	8,089^*^
Contig N50	4.5 Mb^*^	6 kb	0.5 Mb^*^
Genes/Pseudogenes	19,181^*^	13,081	—
Busco	96% complete, 92.5% single, 3.5% duplicate, 0.8% fragmented, 3.3% missing^*^	92.4% complete, 3.2% fragmented	—
Percent masked	43.25%^*^	60%	—
Read N50	8,248	—	—
Read coverage	63	175	96^*^
Scaffolds	8,066^*^	36,095	297^*^
Scaffold N50	48.5 Mb^*^	478 kb	51.1 Mb^*^
Fraction of the genome that was unplaced	3.3%^*^	—	3.6%^*^

Experimental cytometric reports of *L. salmonis* genome size range from ∼567 Mb (Gregory)—1,500 Mb (Wyngaard *et al.* 2022). Annotation metrics and BUSCO scores were reported from GCF_016086655.2 (NCBI), which was updated to remove contamination sequences for GCF_016086655.3.

* Reported by NCBI, “—” not known

**Fig. 1.**
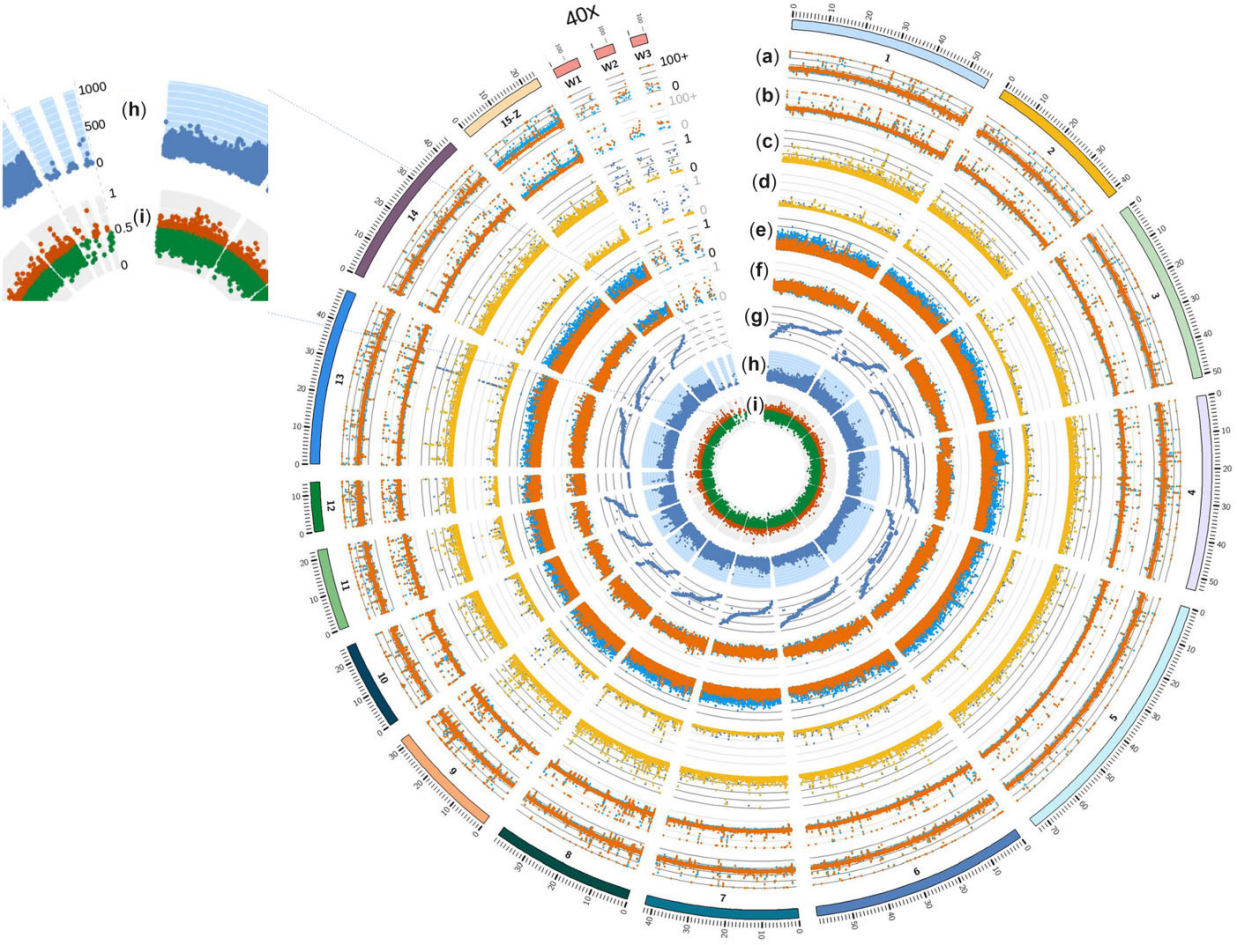
Circos plot of salmon lice genome assembly. Linkage groups with marks every million base-pairs were drawn on the outer edge of the Circos plot. Linkage group 15 was labeled as 15-Z to emphasize that LG15 is the sex-chromosome and that the sex-determination system is ZW. The 3 largest scaffolds from the W-chromosome are shown with a 40x magnification and with marks every 100 kb. They are labeled W1-3. a) The average depth of coverage of the Atlantic subspecies samples in 10 kb windows, blue for the males (on 15-z, the top points) and orange for the females (on 15-z, the bottom points). The maximum coverage displayed is 100x. b) The same as A, except for the Pacific subspecies samples. c) The average ratio of missing genotypes relative to other genotypes of the Atlantic subspecies in 10 kb windows, blue for males (on W1, the top points) and yellow for females (on W1, the bottom points). d) The same as C, except for the Pacific subspecies samples. e) The average ratio of heterozygous genotypes relative to the other genotypes of the Atlantic subspecies in 10 kb windows, blue for males (on 15-z, the top points) and orange for females (on 15-z, the bottom points). f) The same as e, except for the Pacific subspecies samples. g) Marey map of the genetic map (Danzmann *et al.* 2019) used to place contigs onto chromosomes. h) The number of SNPs within 10 kb windows. The white axis lines represent increments of 100. i) The ratio of repetitive elements within 10 kb windows. Orange points are greater than 0.5 (top half). A magnified insert is shown to display the *y*-axis units.

We observed similarly high levels of repetitive elements within the genome as previously reported (Fig. 1, Supplementary Fig. 1, Skern-Mauritzen *et al.* 2021). The salmon louse genome size has previously been estimated between 567 Mb (Gregory) and 1.5 Gb (preprint: Wyngaard *et al.* 2022). Wyngaard *et al.* (2022), explored multiple explanations for the discrepancy between the ∼650 Mb genome size of most salmon louse genome assemblies (Table 1) and the estimate of 1.5 Gb from cytometric data, but suggested that the most likely source was repetitive elements being collapsed into fewer copies in the genome assemblies.

**Fig. 2.**
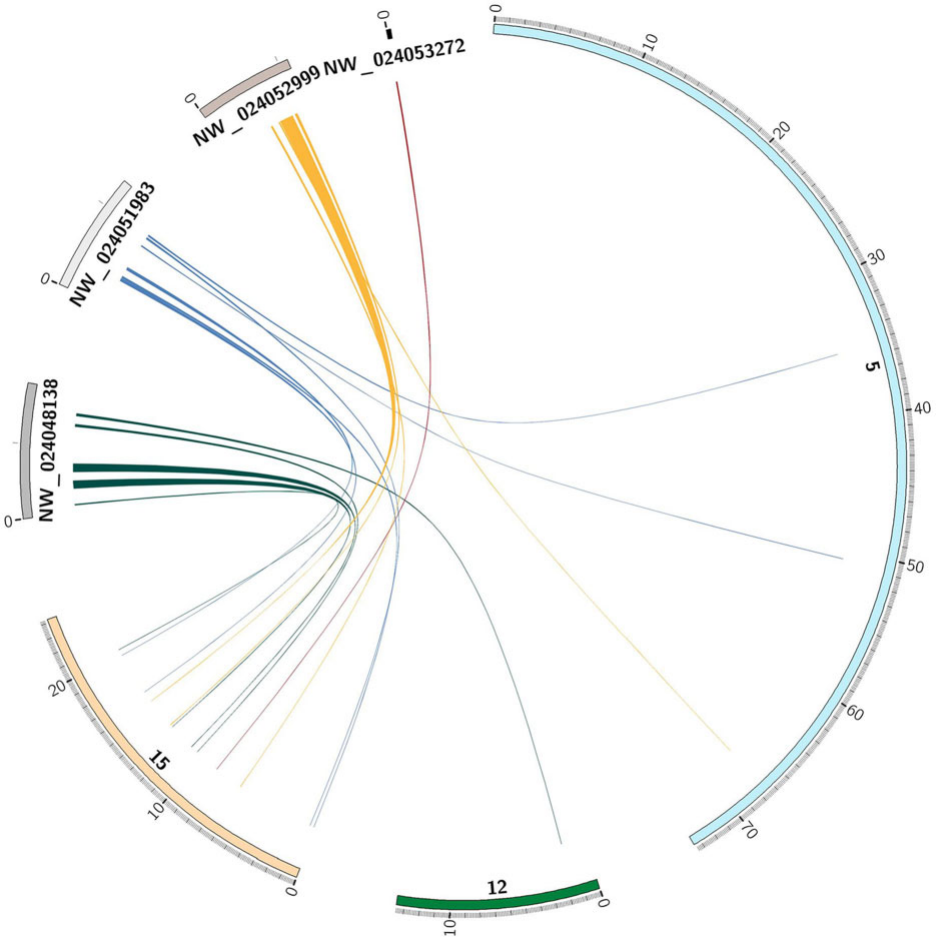
Circos plot of proteins on W-chromosome scaffolds aligned to the remainder of the genome. Four scaffolds composing a large portion of the known W-chromosome were annotated with protein-coding genes. The proteins with a single ortholog or paralog in the remainder of the genome are shown with links between them. The scaffolds were magnified 60x in order to display more detail.

With comparisons of 25 Atlantic and 15 Pacific salmon lice, we were able to better understand genomic features that have previously been identified. In particular, we were able demonstrate that the entire linkage group 15 (the Z-chromosome) has an uneven coverage pattern, with female read coverage half that of males (Fig. 1, a–d, Supplementary Fig. 2). From Danzmann *et al.* (2019), we know that a large number of hemizygous segregating SNP markers were detected across all linkage groups in the genome with the majority (54%) of these localized to linkage group 15. In fact, the vast majority of markers (86% across both sexes and 92% in the male) assigned to linkage group 15 had hemizygous segregation, suggesting that the genome patterning in this chromosome would be very mosaic (Danzmann *et al.* 2019). This was also observed in Skern-Mauritzen *et al.* (2021). In addition, we identified several scaffolds with halved coverage compared to the autosomal chromosomal regions in females (labeled as W1-3 in Fig. 1, Supplementary Fig. 2). These scaffolds appear to be completely missing in males (Fig. 1, Supplementary Fig. 3). Again, these regions were previously mentioned in Skern-Mauritzen *et al.* (2021), but we were able to BLAST (Chen *et al.* 2015) annotated genes on these scaffolds and found that most of the protein-coding genes on these scaffolds had high homology (the average protein % identity was 90.97 and ranged from 72.41% to 99.06% identity) to genes on linkage group 15—likely distantly related orthologs (Fig. 2, Supplementary File 1). Based on the broad distribution of distantly related putative orthologs along linkage group 15 (the Z-chromosome) and the read coverage pattern, we suggest that these scaffolds belong to the W-chromosome in females. If this is the case, it would mean the W-chromosome has been extremely reduced in comparison to the Z-chromosome—excluding repetitive sequences to around 705 kb (Supplementary File 1). The majority of these scaffolds do not align to linkage group 15 except using protein sequences. With only a few candidate genes (Supplementary File 1), identifying a sex-determining gene may be simpler in salmon louse than in other ZW sex-determination systems (e.g. snakes, Matsubara *et al.* 2006), or like in chickens, the reduced W-chromosome may point to a dosage mechanism of sex-determination (Hirst *et al.* 2018).

Another genomic feature that was previously observed was the lack of recombination on linkage group 12 (Danzmann *et al.* 2019). One striking feature of linkage group 12, observed from the current study, was the low number of nucleotide variants identified on this linkage group (Fig. 1h, Supplementary Fig. 4). Low nucleotide variation could appear as reduced recombination if there is not enough variation to detect recombination events (e.g., if all recombination events occurred in the first quarter of the chromosome, but no variants were identified in this region, recombination would not be observed even though it occurs). Further investigation will be needed to distinguish between reduced recombination, reduced genetic diversity, or both hypotheses to explain the observations regarding linkage group 12. Interestingly, linkage group 12 has the lowest nucleotide diversity between salmon lice subspecies (Supplementary Fig. 5). We also note the different segregation patterns in Pacific salmon lice (Supplementary Fig. 6), where alternative homozygous alleles are rarely observed; this suggests that we may not fully understand the mechanisms of inheritance in Pacific salmon lice and alternative mechanisms may need to be explored (e.g., hybridogenesis).

While there were more than 57 million nucleotide variants identified before filtering and ∼14 million after filtering (∼2% of the genome) between resequenced genomes, many of these variants were between the Atlantic and Pacific subspecies [using IGV viewer, (Thorvaldsdóttir *et al.* 2013), we observed these differences, e.g. Supplementary Fig. 6]. This is reflected in a PCA analysis where there is a clear distinction between the Atlantic and Pacific subspecies of salmon lice (Supplementary Fig. 7). The Atlantic salmon lice samples had on average lower levels of heterozygous genotypes in 10 kbp windows than Pacific salmon lice (Fig. 1, e and f, Supplementary Fig. 8; Atlantic female average = 0.11, Atlantic male average = 0.18, Pacific female average = 0.25, Pacific male average = 0.24). With the small sample sizes and sampling distribution, caution should be used in extrapolation to the subspecies as a whole, but reduced heterozygous genotypes could be the result of far fewer host numbers of Atlantic Ocean salmon lice and increased inbreeding.

In conclusion, we have updated the salmon louse reference genome. In doing so, we have increased the known gene catalogue of the species, increased the contiguity of the genome, and we were able to further characterize genomic features. We discovered that the W-chromosome is much reduced compared to other chromosomes and that linkage group 12 may have reduced genetic diversity as well as reduced recombination that had previously been observed.

## Data availability

The genome is available in the NCBI database under the following accession number: GCF_016086655.3. The raw reads are available under: SRR12967560. Previously resequenced genomes from another study are available as: SRR1950515, SRR1950516, SRR6913704, SRR6913705, SRR6913706, SRR6913707, SRR6913708, SRR6913709, SRR6913710, SRR6913711, SRR6913712, SRR6913713, SRR6913721, SRR6913722, SRR6913723, SRR6913724, SRR6913725, SRR6913726, SRR6913727, SRR6913728, SRR6913729, SRR6913730, SRR6913737, SRR6913738, SRR6913740, SRR13076813, SRR6913714, SRR6913715, SRR6913716, SRR6913717, SRR6913718, SRR6913719, SRR6913720, SRR6913731, SRR6913732, SRR6913733, SRR6913734, SRR6913735, SRR6913736, SRR6913739. Nucleotide variants in VCF format can be found at: https://doi.org/10.6084/m9.figshare.19026866.v1 (last accessed April 18, 2022). Python scripts are available on github.com (github.com/KrisChristensen/VCFStatistics and github.com/KrisChristensen/NCBIGenomeRepeats; last accessed April 18, 2022).

Supplemental material is available at *G3* online.

## Supplementary Material

jkac087_Supplemental_Figure_1

jkac087_Supplemental_Figure_2

jkac087_Supplemental_Figure_3

jkac087_Supplemental_Figure_4

jkac087_Supplemental_Figure_5

jkac087_Supplemental_Figure_6

jkac087_Supplemental_Figure_7

jkac087_Supplemental_Figure_8

jkac087_Supplemental_File_1

jkac087_Supplemental_Material_Legends
