# Supplementary figures and images for: An update of the salmon louse (Lepeophtheirus salmonis) reference genome assembly
Source: G3 (Bethesda). 2022 Apr 11;12(6):jkac087. doi: 10.1093/g3journal/jkac087 (PMC9157166; doi:10.1093/g3journal/jkac087)

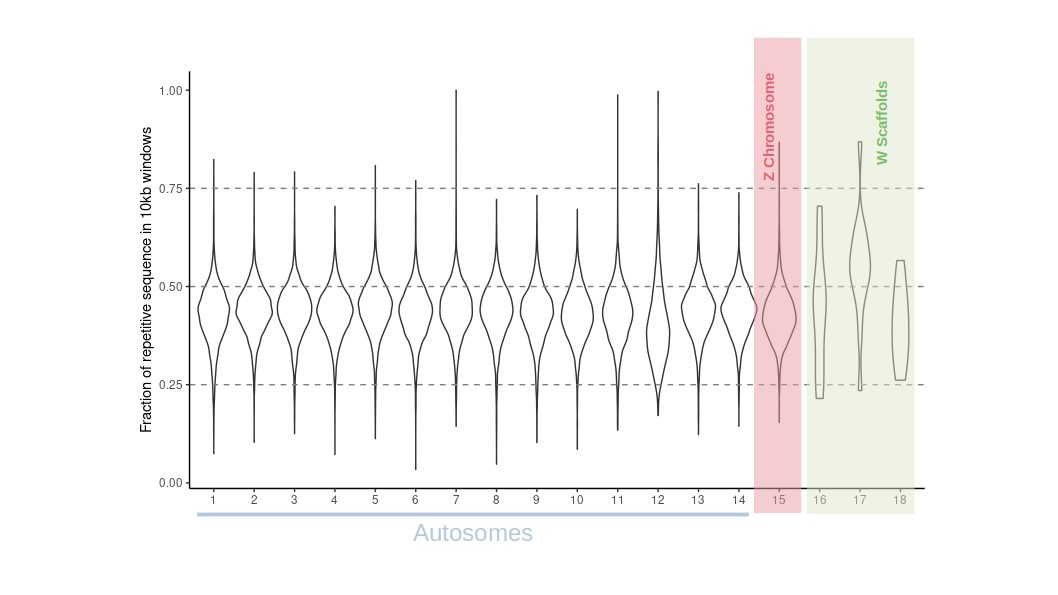

Supplement: jkac087_Supplemental_Figure_1 [file jkac087_supplemental_figure_1.jpeg]

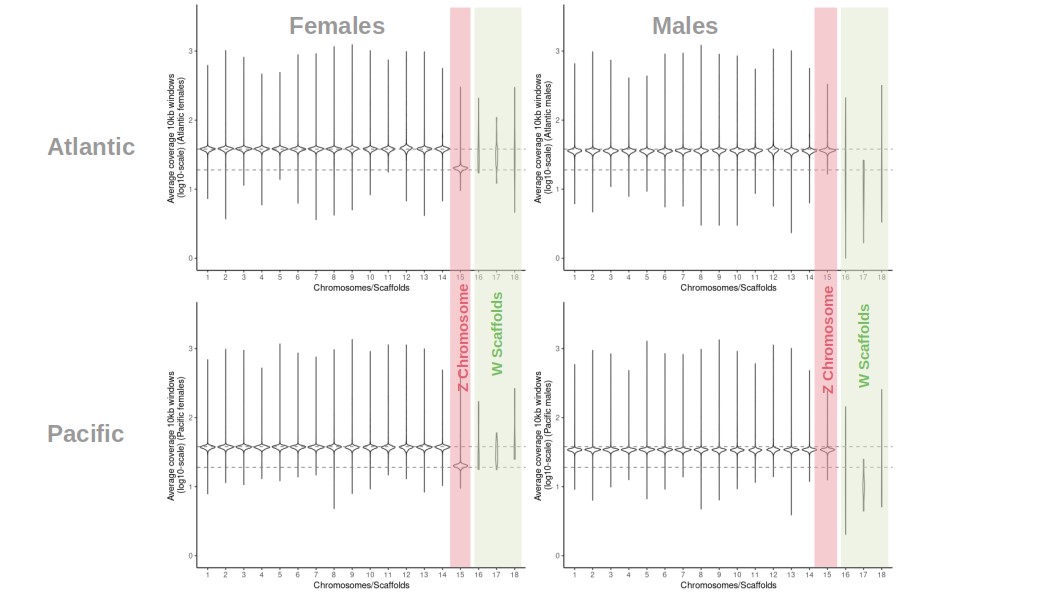

Supplement: jkac087_Supplemental_Figure_2 [file jkac087_supplemental_figure_2.jpeg]

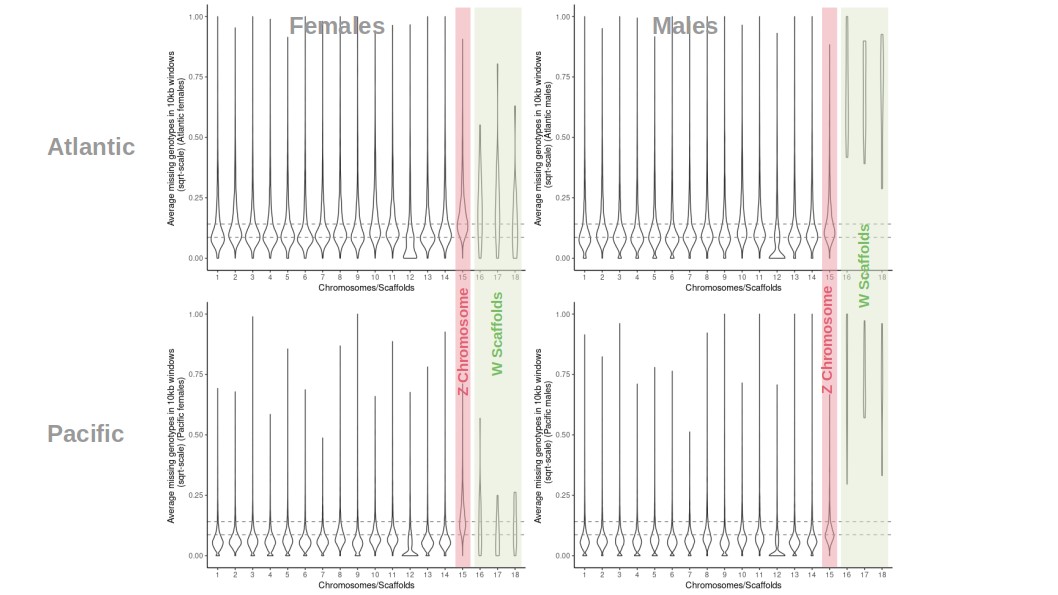

Supplement: jkac087_Supplemental_Figure_3 [file jkac087_supplemental_figure_3.jpeg]

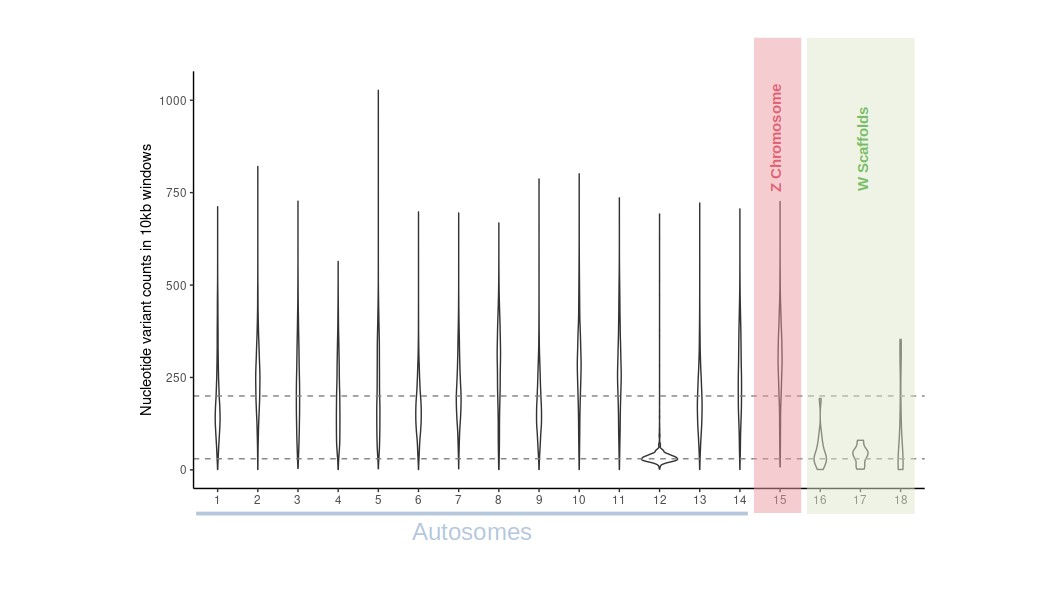

Supplement: jkac087_Supplemental_Figure_4 [file jkac087_supplemental_figure_4.jpeg]

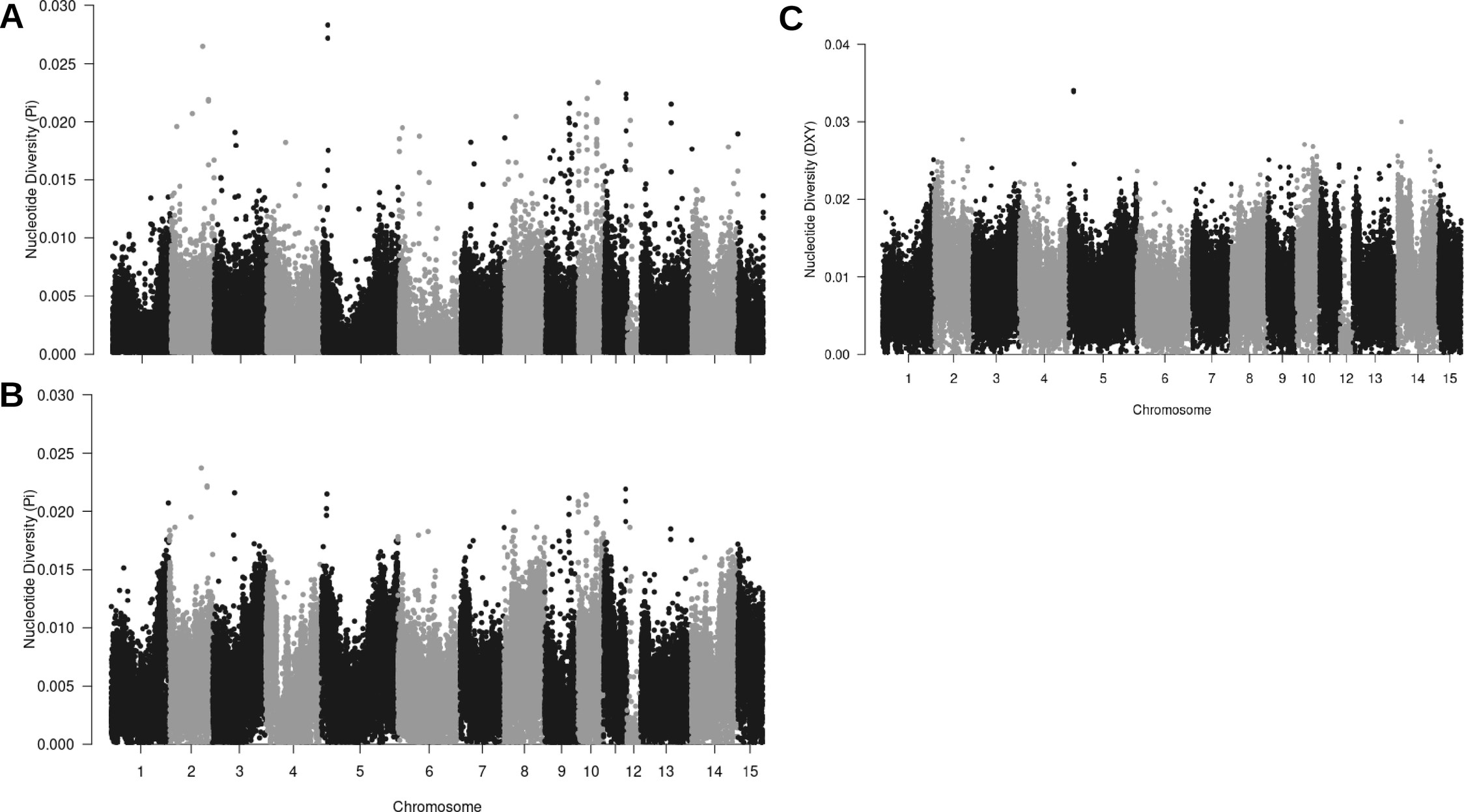

Supplement: jkac087_Supplemental_Figure_5 [file jkac087_supplemental_figure_5.jpeg]

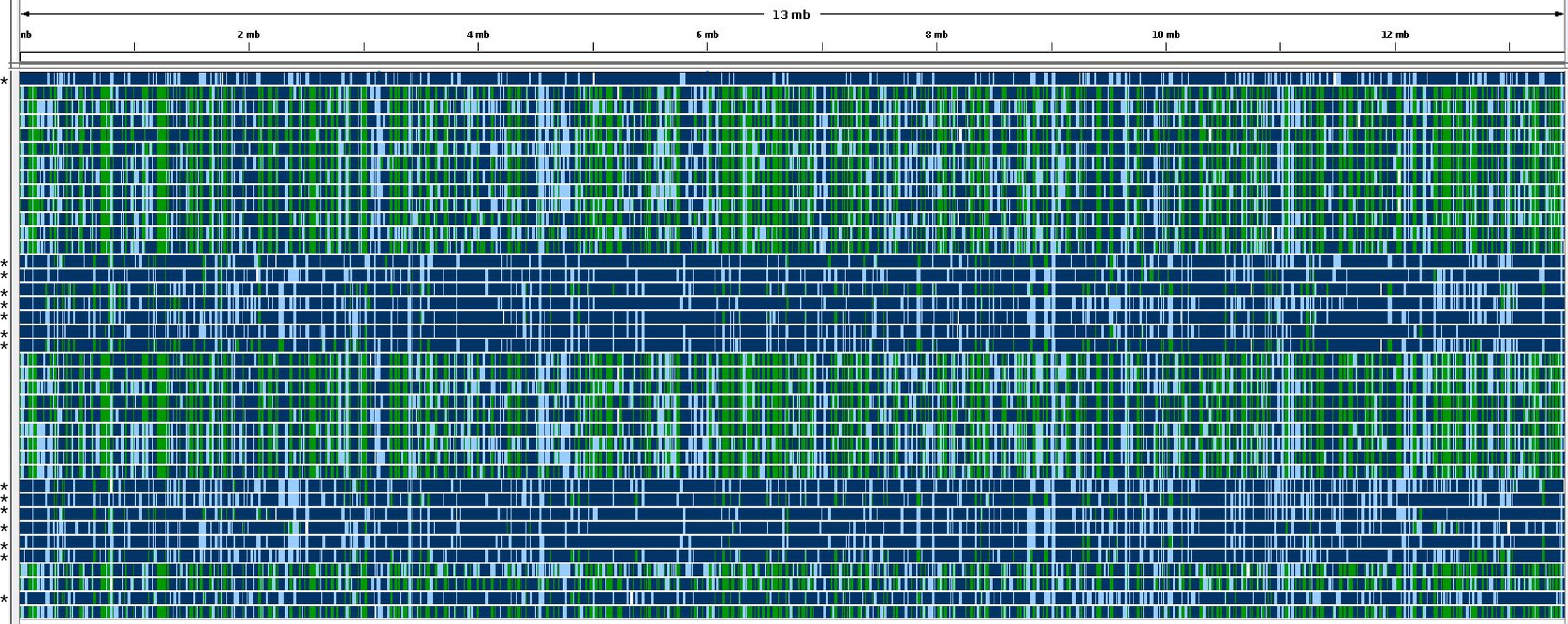

Supplement: jkac087_Supplemental_Figure_6 [file jkac087_supplemental_figure_6.jpeg]

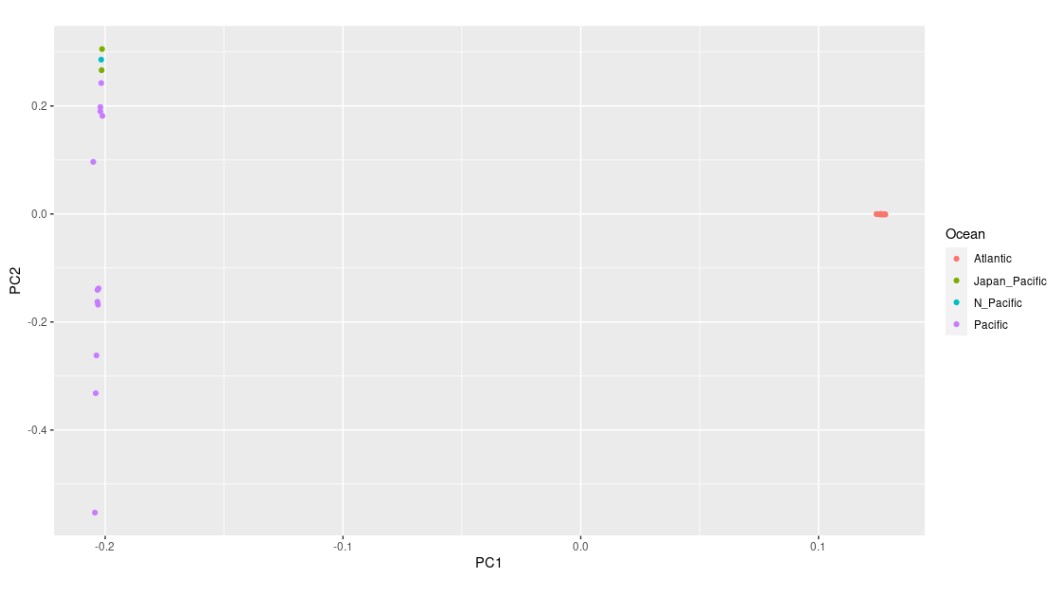

Supplement: jkac087_Supplemental_Figure_7 [file jkac087_supplemental_figure_7.jpeg]

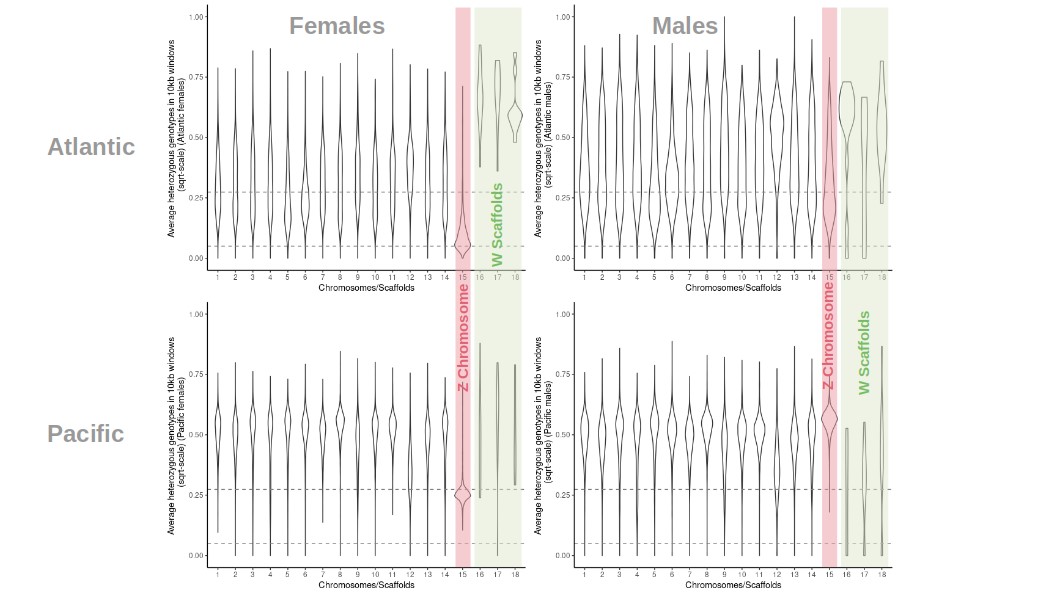

Supplement: jkac087_Supplemental_Figure_8 [file jkac087_supplemental_figure_8.jpeg]
