# Supplementary material for: An update of the salmon louse (Lepeophtheirus salmonis) reference genome assembly
Source: G3 (Bethesda). 2022 Apr 11;12(6):jkac087. doi: 10.1093/g3journal/jkac087 (PMC9157166; doi:10.1093/g3journal/jkac087)
Supplement: jkac087_Supplemental_Material_Legends [file jkac087_supplemental_material_legends.docx]

Supplemental Figure 1. **Fraction of repetitive elements across chromosomes.** A violin plot of the fraction of repetitive sequence in the genome as identified by NCBI for each chromosome (1-15) and for the W-chromosome scaffolds (16-18). Dashed horizontal lines were added for comparison and represent fractions of 0.25, 0.5, and 0.75.

Supplemental Figure 2. **The average genome coverage in 10 kb windows.** Violin plots for categories of salmon louse (Atlantic subspecies, Pacific subspecies, male, and female). This represents the distribution of average coverage of all individuals in a category across all nucleotide variants in 10 kb windows. The X-axis represents the chromosomes (1-15) and the W-chromosome scaffolds (16-18). Dashed horizontal lines were added for comparison and represent coverage depths of 19x and 38x.

Supplemental Figure 3. **The average ratio of missing genotypes in 10 kb windows.** Violin plots for categories of salmon louse (Atlantic subspecies, Pacific subspecies, male, and female). This represents the distribution of average missing genotype ratios (missing genotype count/all genotype calls) of all individuals in a category across all nucleotide variants in 10 kb windows. The X-axis represents the chromosomes (1-15) and the W-chromosome scaffolds (16-18). Dashed horizontal lines were added for comparison and represent 0.0075 and 0.02 missing genotype ratios per 10 kb.

Supplemental Figure 4. **The number of nucleotide variants in 10 kb windows.** Violin plots for the distribution of 10 kb windows with the number of nucleotide variants along chromosomes. The X-axis represents the chromosomes (1-15) and the W-chromosome scaffolds (16-18). Dashed horizontal lines were added for comparison and represent 30 and 200 nucleotide variants per 10 kb window.

Supplemental Figure 5. **Nucleotide diversity within (pi) and between (Dxy) salmon lice subspecies.** A) A Manhattan plot of nucleotide diversity (pi) within Atlantic salmon lice subspecies in 10 kb windows. B) Same as A, except for Pacific salmon lice subspecies. C) Nucleotide diversity between Atlantic and Pacific salmon lice subspecies.

Supplemental Figure 6. **IGV screenshot of linkage group 12.** Genotypes of samples on linkage group 12 viewed in IGV. Each row depicts one salmon louse and each column represents a variant. The variants are dark blue (homologous reference), light blue (heterozygous), and green (homologous alternative). Pacific Ocean salmon lice are shown with an * on the left.

Supplemental Figure 7. **PCA based on SNPs mapped to chromosomes.**  Nucleotide variants passing filtering parameters (variants on unplaced scaffolds were also discarded for this analysis) of individual salmon lice previously published.

Supplemental Figure 8. **The average heterozygous genotype ratios in 10 kb windows.** Violin plots for categories of salmon louse (Atlantic subspecies, Pacific subspecies, male, and female). This represents the distribution of average heterozygous genotype ratios (heterozygous genotype count/all called genotypes) of all individuals in a category across all nucleotide variants in 10 kb windows. The X-axis represents the chromosomes (1-15) and the W-chromosome scaffolds (16-18). Dashed horizontal lines were added for comparison and represent 0.0025 and 0.075 heterozygous genotype ratios per 10 kb window.

Supplemental File 1. **Supplementary data of the contigs/scaffolds with evidence that they map to the salmon louse W-chromosome.** The scaffolds tab has a list of scaffolds with missing genotypes in males, but not in females. The genes tab has a list of protein-coding genes on the W-chromosome scaffolds with known genes. It also has information on homology of these genes to other genomic locations.
